# Botulinum toxin A injection for chronic anal fissure: A retrospective observational study

**DOI:** 10.1097/MD.0000000000048795

**Published:** 2026-05-08

**Authors:** Haci Vural Soyer, Sinan Aslan, Samed Sayar

**Affiliations:** a Department of General Surgery, Mersin City Training and Research Hospital, Mersin, Türkiye.

**Keywords:** botulinum toxin A, chemical sphincterotomy, chronic anal fissure, Dysport®, lateral internal sphincterotomy, minimally invasive treatment, pain relief

## Abstract

Chronic anal fissure (CAF) is a common anorectal disorder associated with severe pain and impaired quality of life. Botulinum toxin A (BTA) injection has emerged as a minimally invasive alternative to lateral internal sphincterotomy; however, data regarding high-dose Dysport® protocols and the clinical relevance of booster injections remain limited. This retrospective single-center observational study was conducted at the General Surgery Clinic of Mersin City Training and Research Hospital. Patients treated for CAF between January 1, 2025, and December 1, 2025, were identified through institutional medical records. Sixty consecutive patients were screened; 10 were excluded because they did not attend follow-up visits, and 50 patients with complete follow-up data were included in the final analysis. All patients received a standardized 75 IU Dysport® injection targeting the internal anal sphincter. At the first follow-up reassessment, a booster injection was considered when either <50% reduction in visual analog scale (VAS) pain score or incomplete fissure healing was present. Pain severity was assessed using the VAS, and fissure healing was evaluated clinically and anoscopically. Outcomes were analyzed. The mean age was 45.8 ± 12.3 years, and 28 patients (56%) were men. The mean VAS pain score decreased from 7.80 ± 0.99 (95% confidence interval [CI]: 7.52–8.08) at baseline to 3.18 ± 0.85 (95% CI: 2.94–3.42) at 1 month and 1.00 ± 0.67 (95% CI: 0.81–1.19) at 3 months. Repeated-measures analysis of variance demonstrated a statistically significant reduction in pain over time (*F*[2, 98] = 1512.68, *P* < .001, partial η^2^ = 0.969). Healing was observed in 34/50 patients (68.0%, 95% CI: 54.2–79.2) at 1 month and 45/50 patients (90.0%, 95% CI: 78.6–95.7) at 3 months. Booster injections were required in 30/50 patients (60.0%, 95% CI: 46.2–72.4). Recurrence was observed in 5/50 patients (10.0%, 95% CI: 4.3–21.4) during follow-up; these patients subsequently underwent lateral internal sphincterotomy. Transient mild incontinence occurred in 3/50 patients (6.0%, 95% CI: 2.1–16.2) and resolved with conservative management. High-dose Dysport® injection appears to be a safe and effective minimally invasive option for the short-term treatment of CAF. A booster-based stepwise strategy may improve outcomes in selected patients before escalation to surgery.

## 1. Introduction

Chronic anal fissure (CAF) is a common anorectal disorder characterized by a painful, non-healing ulcer of the anal canal that significantly impairs the quality of life in the adult population. The pain is typically described as sharp or burning, exacerbated during defecation, and often leads to avoidance behaviors, constipation, and progressive deterioration in overall well-being.^[1]^

The pathophysiology of CAF is multifactorial; however, hypertonicity of the internal anal sphincter represents the central mechanism. Increased resting anal pressure results in reduced anodermal blood flow and localized ischemia, which impairs tissue healing and perpetuates the vicious cycle of pain, ischemia, and sphincter spasm.^[2]^ Current treatment strategies aim to disrupt this pathophysiological cycle.

Initial management typically involves conservative measures, including dietary fiber supplementation and topical pharmacological agents, such as nitric oxide donors or calcium channel blockers, intended to reduce sphincter tone and improve local perfusion.

Nevertheless, these approaches frequently yield suboptimal long-term results and are associated with relatively high recurrence rates. Lateral internal sphincterotomy (LIS) has therefore been regarded as the gold standard treatment for CAF due to its high healing rate; however, the procedure carries a reported risk of permanent fecal incontinence ranging from 5 to 30%, which represents a major clinical concern.^[3,4]^

Botulinum toxin A (BTA) injection has emerged as a minimally invasive alternative to surgical intervention by directly targeting underlying sphincter hypertonicity. BTA inhibits presynaptic acetylcholine release at the neuromuscular junction, thereby inducing a temporary chemical sphincterotomy that reduces resting anal pressure and improves anodermal blood flow, facilitating fissure healing.^[5,6]^

Despite the growing body of evidence supporting botulinum toxin A for CAF, several important gaps remain. Recent evidence has further supported botulinum toxin as a sphincter-preserving option in CAF. A 2025 systematic review and meta-analysis of randomized controlled trials reported healing after the first botulinum toxin injection in 72.7% of patients, with additional benefits from second injections in selected non-responders.^[7]^ Reported outcomes vary substantially according to toxin formulation, injected dose, number of injection sites, and follow-up strategy. In particular, the literature on high-dose Dysport protocols remains limited, and the role of booster injections in routine clinical practice is insufficiently characterized.^[8,9]^ Higher-dose botulinum toxin protocols have been investigated to improve therapeutic outcomes without compromising safety.^[10]^ In a 2017 comparative study, high-dose botulinum toxin was associated with a lower recurrence rate than low-dose treatment, without long-term incontinence, supporting the rationale for dose-intensified protocols in selected patients.^[11]^

Therefore, this study aimed to evaluate the short-term clinical effectiveness and safety of a standardized 75 IU Dysport protocol in adult patients with CAFs refractory to medical therapy. We also aimed to assess pain reduction over time, fissure healing rates, need for booster injections, and frequency of rescue LIS.

## 2. Methods

### 2.1. Study design, patient selection, and ethical approval

**Figure 1.**
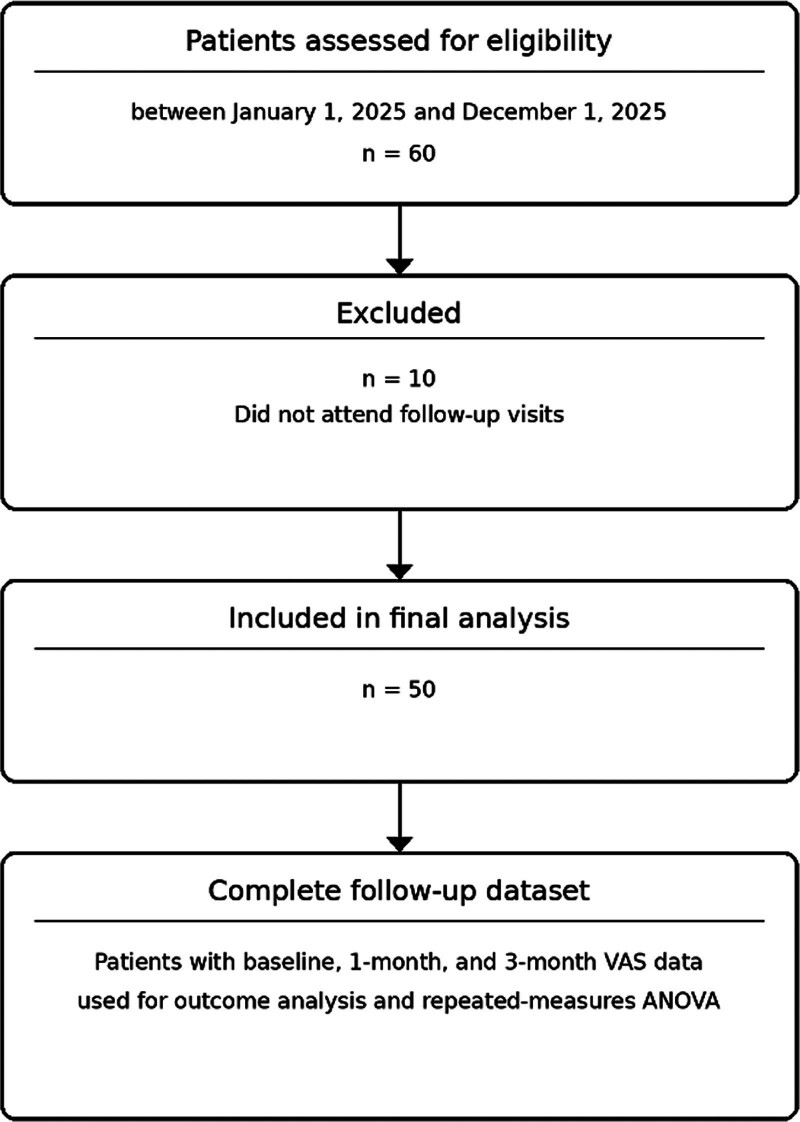
STROBE-style flow diagram of patient selection. Sixty patients with chronic anal fissure were assessed for eligibility between January 1, 2025, and December 1, 2025. Ten patients were excluded because they did not attend follow-up visits. The final analysis included 50 patients with complete follow-up data.

This retrospective single-center observational study was conducted at the General Surgery Clinic of Mersin City Training and Research Hospital. Adult patients treated for CAF between January 1, 2025, and December 1, 2025, were screened through institutional medical records. Sixty consecutive patients were initially identified during the study period. Of these, 10 patients were excluded because they did not attend follow-up visits, and the final analysis included 50 patients with complete follow-up data (Fig. 1).

CAF was defined as a fissure persisting for more than 6 weeks despite at least 8 weeks of conservative medical therapy. Adult patients aged 18 years or older who underwent botulinum toxin A injection for refractory CAF were eligible. Patients with acute anal fissure, inflammatory bowel disease, anal malignancy, pregnancy, previous sphincter surgery, contraindications to botulinum toxin, or incomplete follow-up were excluded. Ethical approval was obtained from the Mersin City Training and Research Hospital Non-Interventional Clinical Research Ethics Committee (approval date: December 24, 2025; decision number: 67). Because of the retrospective design and the use of anonymized patient data, the requirement for informed consent was waived by the ethics committee.

### 2.2. Baseline assessment

Baseline demographic and clinical data were collected, including age, sex, body mass index, and duration of symptoms prior to intervention. Fissure location was classified according to its position relative to the anal canal midline as posterior, anterior, or lateral. Etiology was categorized as chronic constipation–related, postpartum, or post-hemorrhoidectomy.

The presence of a sentinel pile, a common clinical finding in CAF, was recorded. Comorbid conditions, such as hypertension, diabetes mellitus, and the use of anticoagulant or antiplatelet therapy, were documented. Baseline pain intensity was assessed using a visual analog scale (VAS).

### 2.3. Intervention protocol

A commercially available formulation of botulinum toxin A (Dysport®; abobotulinumtoxin A) was used. Each vial was reconstituted with 2.5 mL of sterile saline, according to institutional routine practice, and a total dose of 75 IU was administered at the initial session. To facilitate precise intramuscular injection into the internal anal sphincter, a 30-gauge, 13-mm needle was used. The injection volume was meticulously controlled at 0.4 mL per site, corresponding to approximately 25 IU of Dysport® per injection point, to achieve localized muscle relaxation.

In standard cases, the dose was equally distributed across 3 injection sites: 25 IU was injected at the 3 o’clock position, 25 IU at the 9 o’clock position, and 25 IU at the fissure edge. In one patient with distinct anatomical considerations, injections were administered at the 6 o’clock position, the 12 o’clock position, and the fissure edge. All procedures were performed in an outpatient setting under topical perianal anesthesia.

To minimize patient discomfort, topical anesthesia with 5% lidocaine cream was applied to the perianal region prior to injection.

### 2.4. Booster injection and concomitant therapy

A booster injection was considered at the first follow-up reassessment in patients who met either of the following criteria: <50% reduction in VAS pain score compared with baseline or incomplete fissure healing, defined as the absence of epithelial closure on clinical and anoscopic examination. This decision was made according to the predefined clinical treatment algorithm used in the unit. A booster injection of 20 IU Dysport® was administered to the same injection sites to enhance the initial chemodenervation effect.

All patients were advised to follow a high-fiber diet and received supportive conservative therapy. In addition, patients were treated with calcium dobesilate and a topical vasodilator regimen consisting of either 0.2% diltiazem or 0.4% glyceryl trinitrate, according to routine clinical practice. These adjunctive treatments were not randomized, and the choice of topical therapy was based on clinical preference and tolerability.

### 2.5. Follow-up and outcome measures

Patients were evaluated at 4, 12, and 24 weeks following the initial intervention. Clinical assessments and symptom evaluations were performed at each visit. At the 12-week visit, anoscopy was performed to objectively confirm fissure healing, defined as complete epithelialization of the fissure.

An additional telephone follow-up was performed at 6 months to assess long-term symptom control, recurrence, and patient satisfaction.

### 2.6. Statistical analysis

Statistical analyses were performed using IBM SPSS Statistics for Windows, version 26.0 (IBM Corp., Armonk). Continuous variables are presented as mean ± standard deviation, and categorical variables are presented as numbers and percentages. Ninety-five percent confidence intervals (95% CIs) were calculated for key outcome measures. Changes in VAS pain scores across follow-up visits were evaluated using repeated-measures analysis of variance (ANOVA). Statistical significance was set at *P* < .05.

## 3. Results

**Figure 2.**
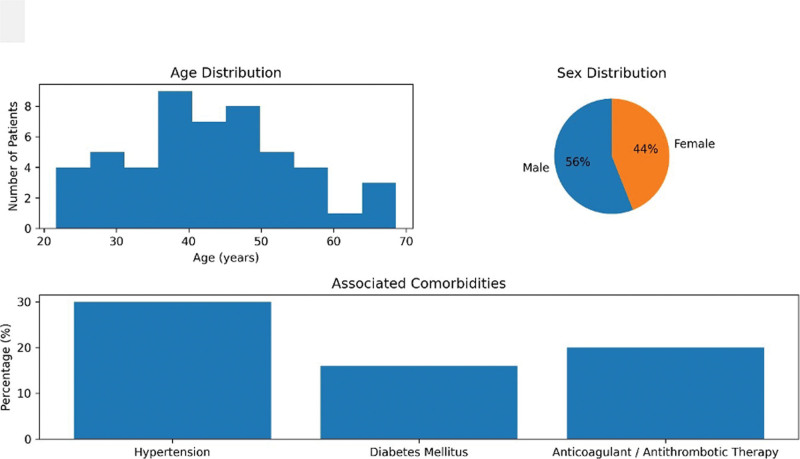
Baseline demographic and clinical characteristics of the study population. This figure summarizes the baseline demographic features of the study population, including age distribution, sex distribution, and associated comorbidities.

**Figure 3.**
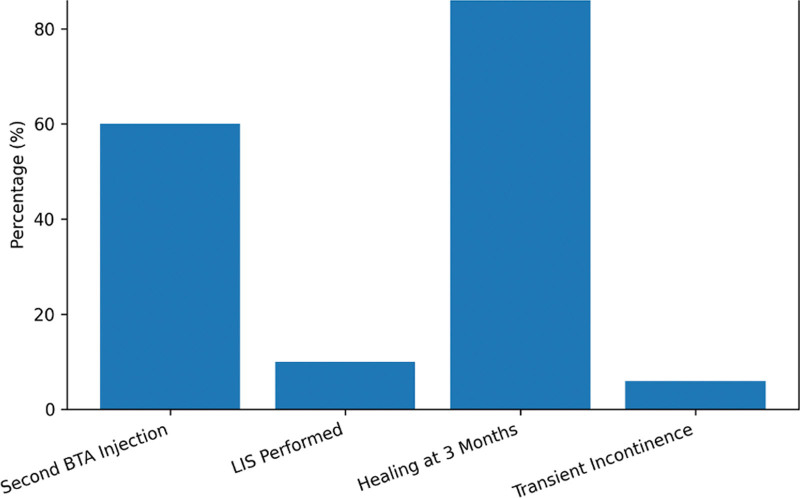
Treatment course and clinical outcomes. This figure shows the proportions of patients requiring a booster botulinum toxin A injection, developing recurrence during follow-up, undergoing lateral internal sphincterotomy, achieving fissure healing at 3 mo, and developing transient mild incontinence.

We analyzed data from 50 patients with CAF treated with 75 IU of BTA. Demographic characteristics, treatment course, and clinical outcomes are summarized in Table 1 and Figure 2. The mean age was 45.8 ± 12.3 years (range, 20–75 years), with 28 male patients (56%) and 22 female patients (44%). All patients presented with a single anal fissure and received an initial 75 IU BTA injection. Because of insufficient clinical response at early follow-up, 30/50 patients (60.0%, 95% CI: 46.2–72.4) required a second (booster) injection of 20 IU BTA. Recurrence was observed in 5/50 patients (10.0%, 95% CI: 4.3–21.4) during follow-up, and these patients subsequently underwent LIS (Fig. 3). Fissure location was posterior in 42 patients (84%), anterior in 7 patients (14%), and lateral in 1 patient (2%).

**Table 1:** Demographic and clinical characteristics of the patients.

Variable	Value
Age (yr)	45.8 ± 12.3 (20–75)
Sex	
Male, n (%)	28 (56)
Female, n (%)	22 (44)
Body mass index (kg/m^2^)	26.8 ± 3.9
Symptom duration (wk)	14 (8–36)
Fissure location	
Posterior, n (%)	42 (84)
Anterior, n (%)	7 (14)
Lateral, n (%)	1 (2)
Fissure etiology	
Chronic constipation, n (%)	40 (80)
Postpartum, n (%)	8 (16)
Post-hemorrhoidectomy, n (%)	2 (4)
Sentinel pile present, n (%)	30 (60)
Comorbidities	
Hypertension, n (%)	15 (30)
Diabetes mellitus, n (%)	8 (16)
Anticoagulant/ antithrombotic therapy, n (%)	10 (20)
Baseline VAS score	7.8 ± 1.2

Values are presented as mean ± SD, median (IQR), or n (%), as appropriate.

SD = standard deviation, VAS = visual analog scale.

Regarding etiology, chronic constipation was identified in 40 patients (80%), postpartum factors in 8 patients (16%), and previous hemorrhoid surgery in 2 patients (4%). A sentinel pile was present in 30 patients (60%). The most common comorbidities were hypertension (30%), diabetes mellitus (16%), and the use of anticoagulant or antiplatelet therapy (20%).

Dysport® was used as the BTA formulation and reconstituted with 2.5 mL of sterile saline. In 49 patients (98%), injections were administered on an outpatient basis with the patient in the knee–elbow position at the 3 and 9 o’clock positions and at the fissure edge. In one patient (2%), injections were applied at the 6 and 12 o’clock positions and the fissure edge due to anatomical considerations. Each injection site received 25 IU of BTA in a total volume of 0.4 mL. All patients were informed about the procedure and received adjunctive medical therapy, including a high-fiber diet, calcium dobesilate, and topical vasodilators.

**Figure 4.**
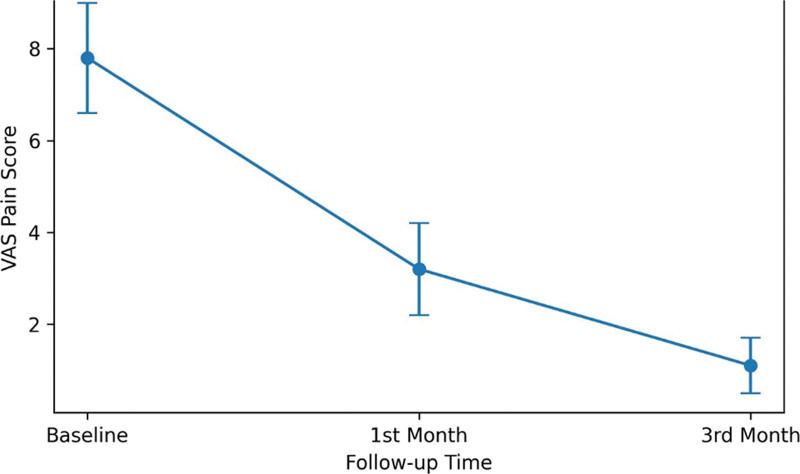
Change in mean VAS pain scores over time. Mean visual analog scale (VAS) pain scores at baseline, 1 mo, and 3 mo are shown. Error bars represent standard deviation.

**Figure 5.**
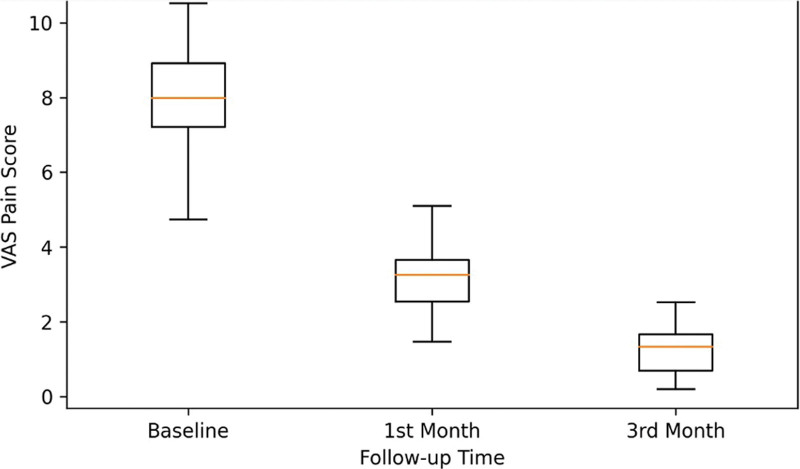
Distribution of VAS scores over time. Box plots show the distribution of visual analog scale (VAS) pain scores at baseline, 1 mo, and 3 mo in the 50 patients with complete follow-up.

VAS pain scores progressively decreased during follow-up (Fig. 4). The mean VAS pain score decreased from 7.80 ± 0.99 (95% CI: 7.52–8.08) at baseline to 3.18 ± 0.85 (95% CI: 2.94–3.42) at 1 month and 1.00 ± 0.67 (95% CI: 0.81–1.19) at 3 months. Repeated-measures ANOVA demonstrated a statistically significant reduction in pain over time (*F*[2, 98] = 1512.68, *P* < .001, partial η^2^ = 0.969). Pairwise comparisons demonstrated significant differences between baseline and 1 month and between baseline and 3 months (*P* < .001 for both). The distribution of VAS scores also narrowed progressively over time (Fig. 5).

Clinical healing was observed in 34/50 patients (68.0%, 95% CI: 54.2–79.2) at 1 month and in 45/50 patients (90.0%, 95% CI: 78.6–95.7) at 3 months. Booster injections were required in 30/50 patients (60.0%, 95% CI: 46.2–72.4). Transient mild incontinence occurred in 3/50 patients (6.0%, 95% CI: 2.1–16.2) and resolved with conservative management. No major complications were observed.

## 4. Discussion

In this retrospective single-center study, a standardized 75 IU Dysport® protocol was associated with marked pain reduction, high short-term healing rates, and a low rate of major complications in patients with CAF refractory to medical therapy. In addition, 60% of patients required a booster injection, supporting the potential clinical relevance of a stepwise treatment strategy in a substantial proportion of cases. However, given the observational design and the absence of a control group, these findings should be interpreted as evidence of short-term effectiveness and clinical feasibility rather than definitive comparative efficacy.

The significant reduction in pain intensity observed in our cohort is one of the most clinically relevant findings of this study. The mean VAS scores decreased from 7.80 at baseline to 1.00 at 3 months, indicating marked symptomatic improvement during the follow-up. These findings are consistent with previous reports demonstrating meaningful improvements in pain and quality of life following BTA treatment for CAF. Importantly, the progressive narrowing of the VAS score distribution over time suggests a more homogeneous treatment response during follow-up, reinforcing the clinical reliability of BTA therapy.

In our cohort, fissure etiology was predominantly related to chronic constipation and postpartum factors, which aligns with reported epidemiological data. The favorable outcomes observed across these etiological subgroups suggest that BTA may be effective in both primary and secondary forms of CAFs. In particular, our results support previous systematic reviews that highlight the benefits of BTA in postpartum fissures, where preservation of continence is of paramount importance and surgical intervention is often deferred.

Compared with surgical sphincterotomy, botulinum toxin A may offer a more favorable safety profile in selected patients, although complete healing rates are generally lower in the literature.^[12]^ The risk of permanent fecal incontinence associated with LIS remains a major concern, especially in younger patients and women. In contrast, the transient mild gas or stool incontinence observed in only 6% of patients in our study resolved with conservative measures, underscoring the reversibility and safety of chemical sphincterotomy. These findings further support the use of BTA as an attractive treatment option for patients who are unsuitable for surgery or who prefer a less invasive approach.

The 75 IU Dysport® protocol used in this study is consistent with emerging evidence suggesting that higher-dose regimens may enhance treatment efficacy without compromising safety. Previous randomized controlled trials and dose–response studies evaluating lower doses of Dysport® or Botox® have reported variable healing rates, often necessitating repeated interventions. The high healing rate and low incidence of complications observed in our study suggest that this dosing strategy is both effective and clinically acceptable.

An important finding of this study was that 30 of the 50 patients (60%) required a booster injection. This relatively high proportion may reflect interindividual variability in baseline sphincter hypertonia, chronicity of disease, or responsiveness to the initial injection. In routine practice, booster treatment was not administered systematically but was guided by early clinical reassessment, particularly in patients with limited symptomatic improvement or incomplete epithelial healing on clinical and anoscopic examination. This stepwise strategy may be especially relevant when sphincter-preserving management is preferred before escalation to surgery. However, the potential contribution of booster injection to the favorable short-term outcomes observed in our cohort should be interpreted with caution, given the absence of a control group and the concurrent use of adjunctive therapies. Taken together, these findings support the practical feasibility of a staged non-surgical treatment pathway in selected patients with CAF.

In our cohort, recurrence was observed in 5 of 50 patients (10%) at 6 months, and these patients subsequently underwent LIS, indicating that surgical intervention remained necessary in a limited subset of patients despite initial BTA-based management. This observation is in line with contemporary long-term data suggesting that persistent or recurrent fissures after botulinum toxin frequently require additional intervention, including repeat injection or surgery, despite the short-term effectiveness of chemical sphincterotomy.^[13]^ The observed recurrence rate is consistent with previous reports evaluating the mid-term efficacy of botulinum toxin A.^[14]^ Furthermore, the absence of serious adverse events and the low incidence of transient complications are consistent with previously published real-world data involving large patient registries, reinforcing the generalizability of our findings to routine clinical practice.

This study has several limitations. First, its retrospective single-center design introduces a risk of selection bias and limits generalizability. Second, there was no control group, precluding direct comparison with alternative medical or surgical treatments. Third, all patients received concomitant conservative therapy, including dietary measures, calcium dobesilate, and topical vasodilators, and these adjunctive treatments were not randomized; therefore, the independent effect of botulinum toxin A could not be isolated with certainty. Fourth, 10 of the 60 initially screened patients were excluded because they did not attend follow-up visits, potentially introducing attrition bias. Fifth, objective physiological measurements such as anal manometry were not available; therefore, mechanistic interpretation of sphincter relaxation could not be directly assessed. Sixth, no multivariable analysis or adjustment for potential confounders was performed because the sample size and the number of outcome events were limited. Finally, the follow-up period was relatively short and may have underestimated long-term recurrence despite the addition of a 6-month telephone follow-up assessment.

Future research should focus on identifying predictors of response to BTA therapy, optimizing dosing regimens and injection techniques, and directly comparing BTA with surgical and emerging treatment modalities in randomized controlled trials. In the context of value-based healthcare, comprehensive cost effectiveness analyses that incorporate both direct medical costs and indirect costs related to productivity loss and quality-of-life impairment are also warranted. Moreover, targeted studies evaluating specific etiological subgroups may further refine treatment algorithms and enable more individualized management of CAFs. Prospective randomized studies are required to confirm causality and further define the optimal role of botulinum toxin A in the management of CAFs.

## 5. Conclusion

High-dose Dysport® injection appears to be a safe and effective minimally invasive option for CAF in the short term. A booster-based stepwise strategy may improve outcomes in selected patients before escalation to surgery. However, prospective controlled studies with longer follow-up are needed to confirm these findings and to better define the independent contribution of botulinum toxin A.

## Author contributions

**Conceptualization:** Haci Vural Soyer, Sinan Aslan.

**Data curation:** Haci Vural Soyer, Sinan Aslan, Samed Sayar.

**Formal analysis:** Haci Vural Soyer.

**Methodology:** Haci Vural Soyer, Sinan Aslan.

**Writing – original draft:** Haci Vural Soyer.

**Writing – review & editing:** Haci Vural Soyer, Sinan Aslan, Samed Sayar.

## References

[1] Sahebally SM Meshkat B Walsh SR Beddy D. Botulinum toxin injection vs topical nitrates for chronic anal fissure: an updated systematic review and meta-analysis of randomized controlled trials. Colorectal Dis. 2018;20:6–15. 29166553 10.1111/codi.13969

[2] Hemmati H Soltany S Toussy J Salehi D Toosi P. Therapeutic properties of botulinum toxin on chronic anal fissure treatment and the patient factors role. J Family Med Prim Care. 2020;9:1562–7. 32509650 10.4103/jfmpc.jfmpc_944_19 PMC7266196

[3] Wollina U. Pharmacological sphincterotomy for chronic anal fissures by botulinum toxin A. J Cutan Aesthet Surg. 2008;1:58–63. 20300345 10.4103/0974-2077.44160 PMC2840903

[4] Nelson RL Chattopadhyay A Brooks W Platt I Paavana T Earl S. Operative procedures for fissure in ano. Cochrane Database Syst Rev. 2011;2011:CD002199. 22071803 10.1002/14651858.CD002199.pub4 PMC7098462

[5] Amorim H Santoalha J Cadilha R Festas MJ Barbosa P Gomes A. Botulinum toxin improves pain in chronic anal fissure. Porto Biomed J. 2017;2:273–6. 32258781 10.1016/j.pbj.2017.04.005 PMC6806753

[6] Brisinda G Vanella S Crocco A Maria G. Type A botulinum toxin treatment for chronic anal fissure. Int J Colorectal Dis. 2012;27:1543–5. 22350272 10.1007/s00384-012-1441-7

[7] Thippeswamy KM Gruber M Abdelaziz H Abdel-Dayem M. Efficacy and safety of botulinum toxin injection in the management of chronic symptomatic anal fissure: a systematic review and meta-analysis of randomized controlled trials. Tech Coloproctol. 2025;29:44. 39786616 10.1007/s10151-024-03087-y

[8] Jost WH Schimrigk K. Therapy of anal fissure using botulin toxin. Dis Colon Rectum. 1994;37:1321–4. 7995166 10.1007/BF02257805

[9] Bobkiewicz A Francuzik W Krokowicz L. Botulinum toxin injection for treatment of chronic anal fissure: is there any dose-dependent efficiency? A meta-analysis. World J Surg. 2016;40:3064–72. 27539490 10.1007/s00268-016-3693-9 PMC5104788

[10] Brisinda G Maria G Sganga G Bentivoglio AR Albanese A Castagneto M. Effectiveness of higher doses of botulinum toxin to induce healing in patients with chronic anal fissures. Surgery. 2002;131:179–84. 11854696 10.1067/msy.2002.119314

[11] Ravindran P Chan DL Ciampa C George R Punch G White SI. High-dose versus low-dose botulinum toxin in anal fissure disease. Tech Coloproctol. 2017;21:803–8. 29080958 10.1007/s10151-017-1700-2

[12] Maria G Brisinda G Bentivoglio AR Cassetta E Gui D Albanese A. Botulinum toxin injections in the internal anal sphincter for the treatment of chronic anal fissure: long-term results after two different dosage regimens. Ann Surg. 1998;228:664–9. 9833804 10.1097/00000658-199811000-00005 PMC1191571

[13] Aboelmaaty S Cheng Ng J Gupta A. Long-term outcomes of botulinum toxin in the treatment of chronic anal fissures: a stepwise approach to managing recurrent and persistent cases. Dis Colon Rectum. 2026;69:608–16. 41489292 10.1097/DCR.0000000000004034

[14] Minguez M Herreros B Espi A. Long-term follow-up (42 months) of chronic anal fissure after healing with botulinum toxin. Gastroenterology. 2002;123:112–7. 12105839 10.1053/gast.2002.34219

